# Capillary hemangioma of the breast presenting as a new mass on screening mammography: A case report with imaging-pathology correlation

**DOI:** 10.1016/j.radcr.2026.08.005

**Published:** 2026-10-09

**Authors:** Romuald Ferre, Anne Bartels, Cherie M. Kuzmiak

**Affiliations:** a Division of Breast Imaging, Department of Radiology, UNC School of Medicine, Physicians' Office Building, Room 118, 170 Manning Dr, Chapel Hill, NC 27599, USA; b Department of Pathology and Laboratory Medicine, UNC School of Medicine, Brinkhous-Bullitt Building, 160 Medical Drive, Campus Box #7525, Chapel Hill, NC 27599-7525, USA

**Keywords:** Capillary hemangioma, Breast hemangioma, Screening mammography, Breast ultrasound, Vascular breast lesion, Imaging-pathology concordance

## Abstract

Capillary hemangioma of the breast is a rare benign vascular lesion that may be detected as a new mass at screening mammography. We report a 74-year-old asymptomatic woman with a newly developed 0.7-cm mass in the posterior left breast. Diagnostic mammography showed an oval, equal-density mass with partially obscured margins, and targeted ultrasound demonstrated a 0.6-cm oval, circumscribed, hypoechoic superficial mass with posterior acoustic enhancement and limited internal Doppler flow associated with a feeding vessel. Because the finding was new and indeterminate, it was assessed as BI-RADS 4A and sampled by ultrasound-guided core needle biopsy. Histologic examination showed a well-circumscribed proliferation of capillary-sized, erythrocyte-containing vessels lined by a single layer of bland, flattened endothelial cells, without cytologic atypia or mitotic activity. D2-40 highlighted background lymphatic vessels but was negative in the lesional channels. No histologic features of angiosarcoma were identified in the sampled tissue. Imaging and pathology were concordant; no surgical excision was performed, and the patient returned to routine annual screening. This case illustrates that interval development may justify biopsy despite relatively benign morphology and that a small capillary hemangioma with benign concordant pathology in an asymptomatic older patient may be managed conservatively while recognizing the sampling limitations of core biopsy.

## Introduction

Breast hemangiomas are uncommon benign vascular tumors, accounting for less than 0.1% of benign breast tumors [1,2]. They are classified as capillary, cavernous, or mixed and may be detected incidentally or during evaluation of a clinical or imaging finding. Their mammographic and sonographic appearances are variable and can overlap with those of other benign and malignant breast masses [[1], [2], [3]].

The principal pathologic differential diagnosis is low-grade angiosarcoma. Because core needle biopsy samples only part of a lesion, histologic findings must be interpreted together with lesion size, interval change, imaging features, and clinical context. We present a screening-detected, sub centimeter capillary hemangioma in a 74-year-old woman that was biopsied because it was newly developed despite otherwise relatively benign morphology. The case emphasizes how interval development can justify biopsy and how sampling-aware imaging-pathology concordance can support conservative management after a benign core biopsy.

## Case presentation

A 74-year-old asymptomatic woman underwent routine screening mammography. Comparison with a prior examination performed 12 months earlier showed a new mass in the posterior left breast at the 12 o'clock position (Fig. 1). An anterior clip marker inferior and medial to the nipple corresponded to a prior benign image-guided biopsy and did not represent prior breast surgery.

**Fig. 1:**
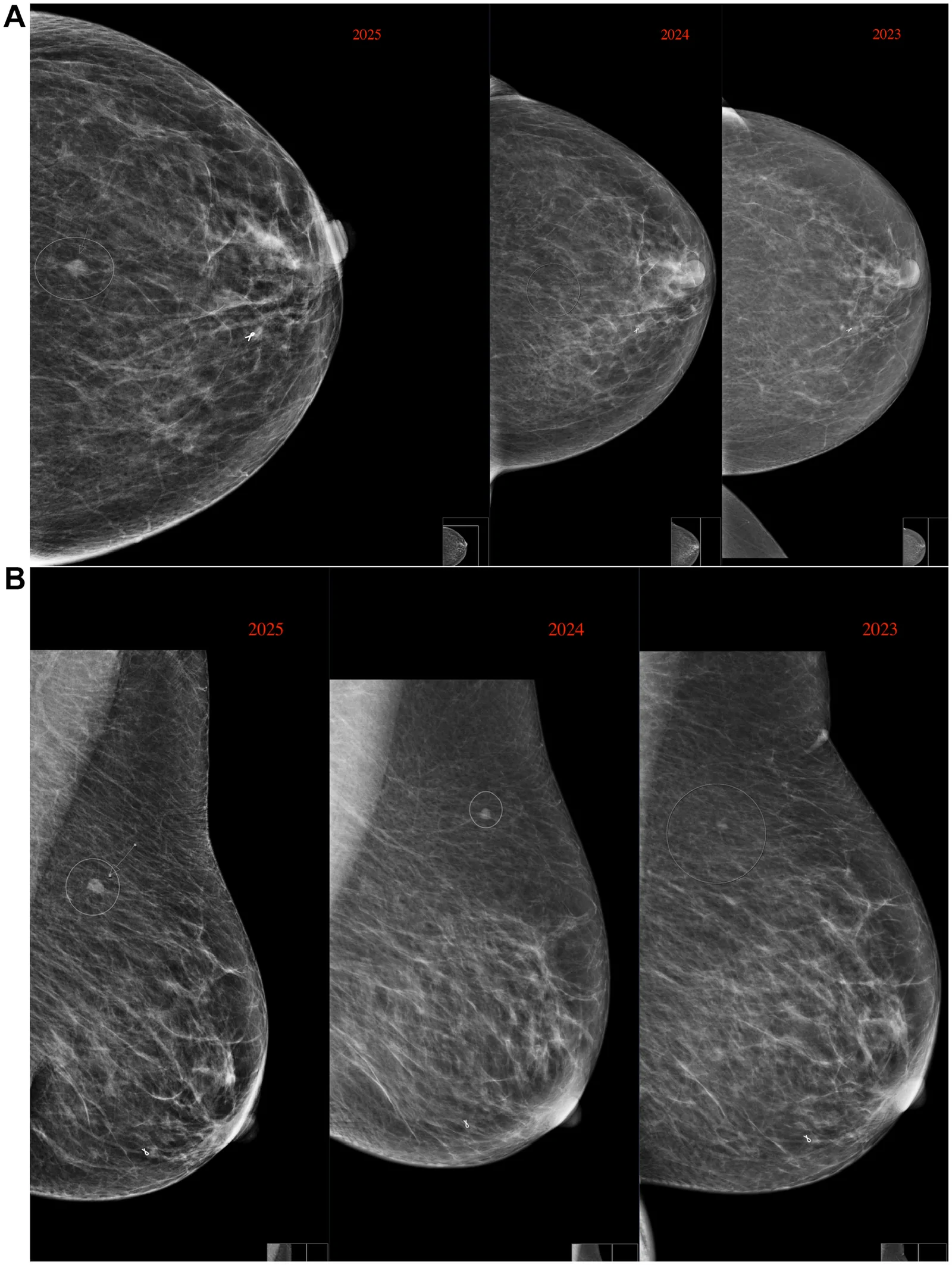
Serial comparison mammograms of the left breast. (A) Craniocaudal views from 2025, 2024, and 2023 demonstrate a newly developed 0.7-cm oval, equal-density mass with partially obscured margins in the posterior breast at the 12 o'clock position (circle and arrow). (B) Mediolateral oblique views from the same examinations confirm interval development of the posterior mass. The anterior clip marker inferior and medial to the nipple is from a prior benign image-guided biopsy and does not represent prior breast surgery.

Diagnostic mammography demonstrated a 0.7-cm oval, noncalcified, equal-density mass with partially obscured margins in the posterior breast. The contralateral breast was unremarkable. Targeted ultrasound showed a 0.6-cm oval, hypoechoic, circumscribed, parallel superficial mass with posterior acoustic enhancement in the upper inner breast (Fig. 2). Color Doppler demonstrated limited internal flow associated with a feeding vessel. Although the morphology was not overtly malignant, the interval development of the mass remained indeterminate. The lesion was assessed as BI-RADS 4A, and ultrasound-guided core needle biopsy was performed (Fig. 3).

**Fig. 2:**
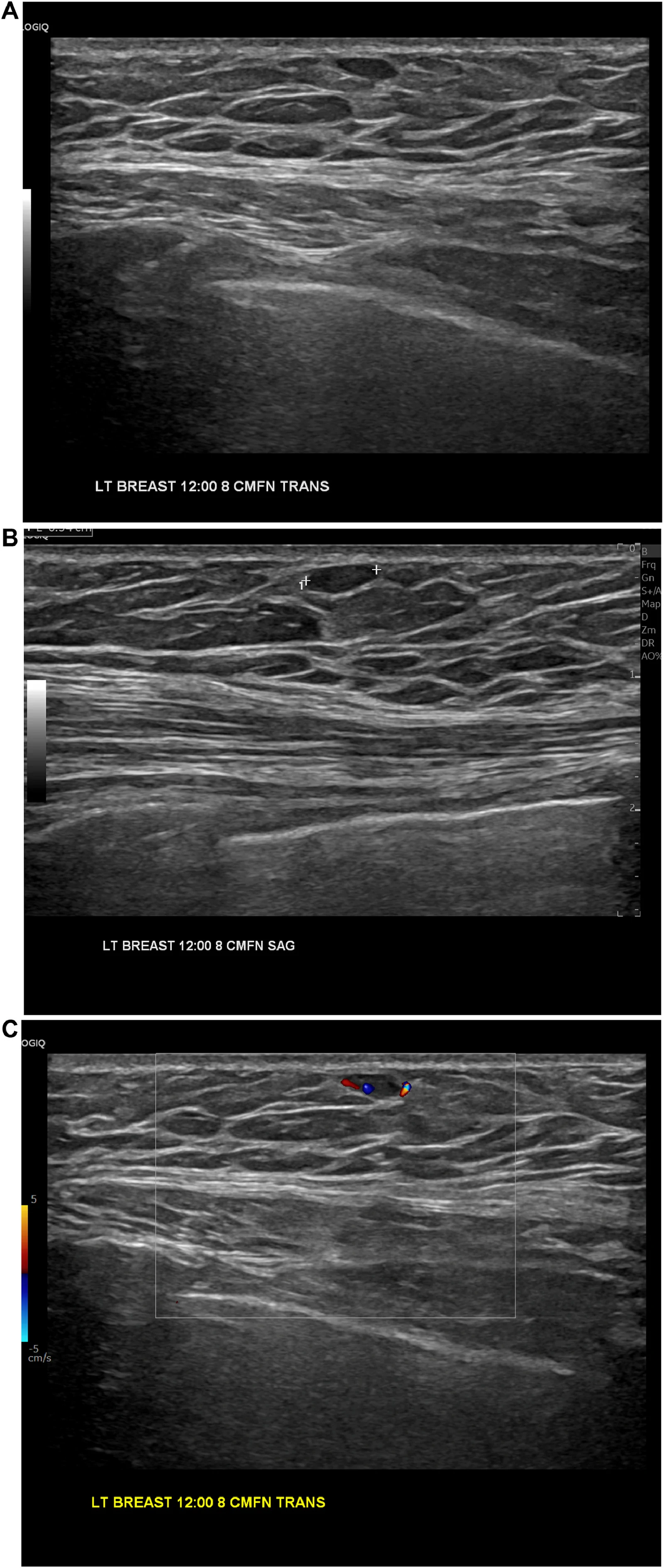
Targeted ultrasound of the left breast at 12 o'clock, 8 cm from the nipple. (A) Transverse and (B) sagittal grayscale images show a 0.6-cm oval, hypoechoic, circumscribed, parallel superficial mass with posterior acoustic enhancement. (C) Color Doppler demonstrates limited internal flow associated with a feeding vessel.

**Fig. 3:**
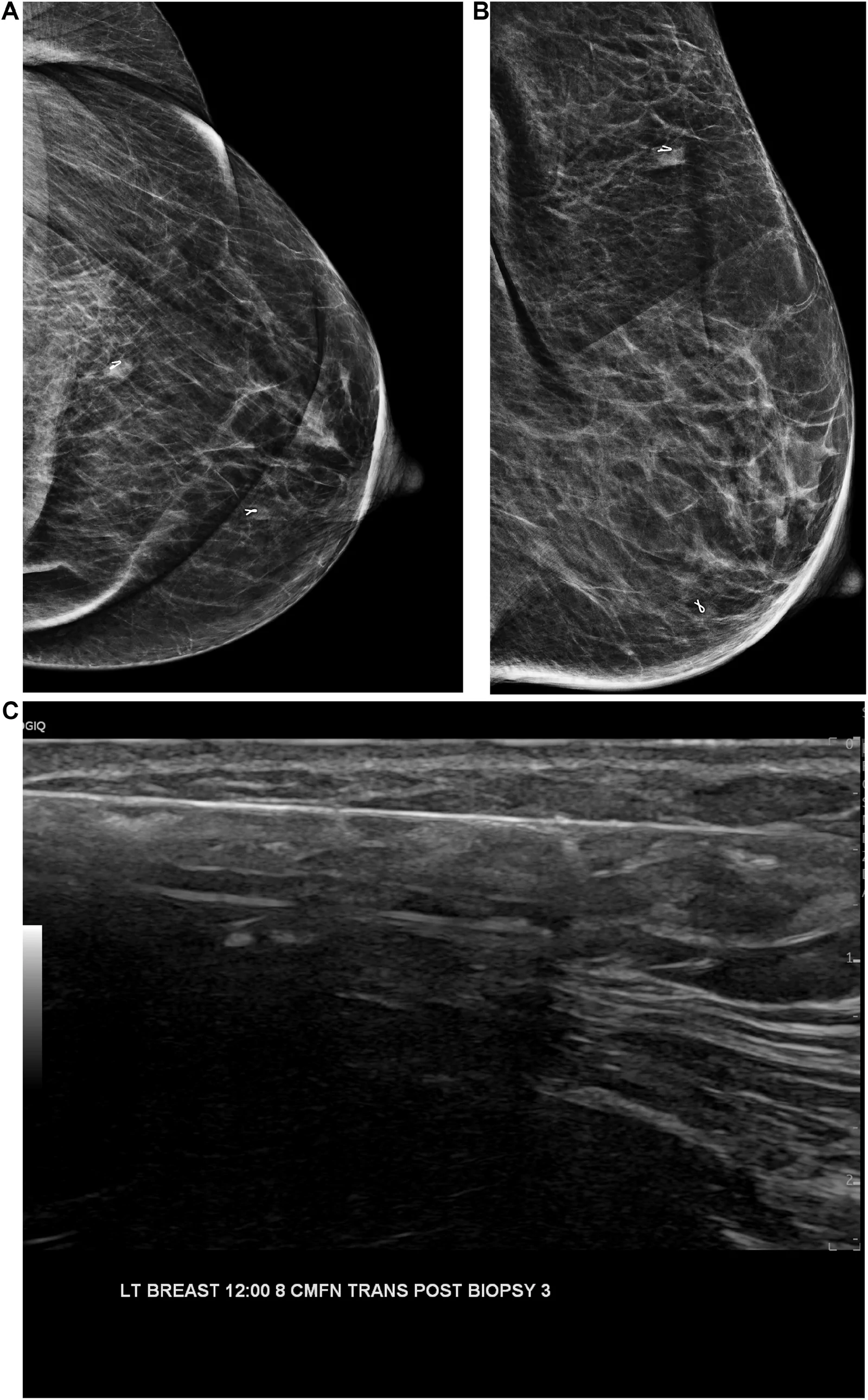
Postbiopsy imaging. (A) Craniocaudal and (B) mediolateral oblique postbiopsy mammograms demonstrate satisfactory deployment of the biopsy marker at the site of the sampled posterior breast mass. The separate anterior marker inferior and medial to the nipple corresponds to a prior benign image-guided biopsy. (C) Postbiopsy ultrasound demonstrates the biopsy needle traversing the targeted superficial mass.

## Pathology

Histologic sections showed a well-circumscribed, bland vascular proliferation within fibroadipose tissue, composed of lobules of capillary-sized, erythrocyte-containing vessels (Fig. 4A). At higher magnification, the vessels were lined by a single layer of bland, flattened endothelial cells without cytologic atypia or mitotic activity (Fig. 4B). D2-40 highlighted background lymphatic vessels but was negative in the lesional vascular channels (Fig. 4C). No additional endothelial immunostains, including CD31, ERG, or CD34, were performed because blood-vascular differentiation was apparent on hematoxylin and eosin sections from the erythrocyte-containing lumina and endothelial lining. The findings supported a diagnosis of benign capillary hemangioma, with no histologic features of angiosarcoma identified in the sampled tissue.

**Fig. 4:**
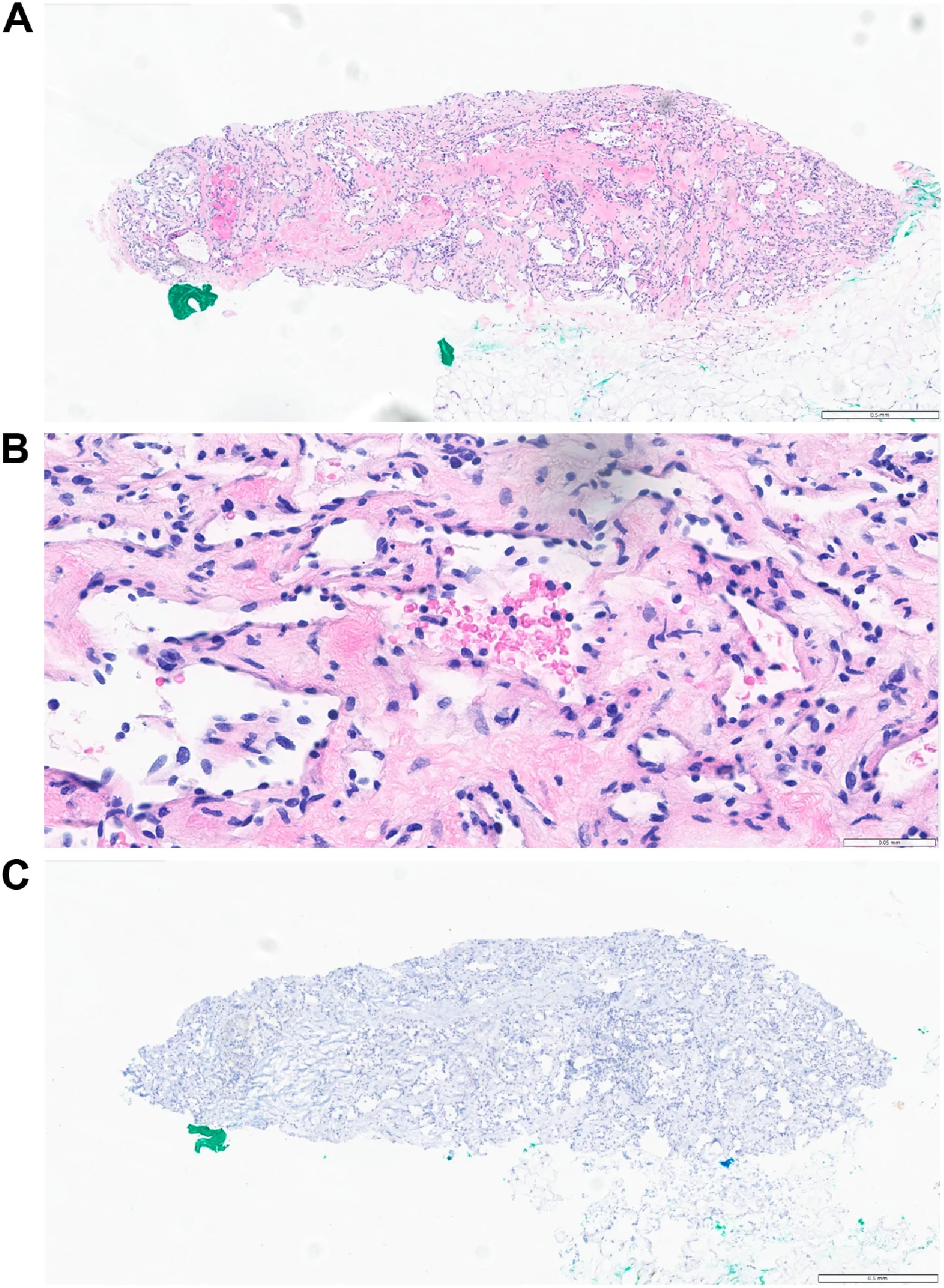
Histopathology of the breast lesion. (A) Low-power hematoxylin and eosin (H&E) image shows a well-circumscribed proliferation of small vascular channels within fibroadipose tissue. (B) Higher-power H&E image obtained with a 40× objective (400× total magnification) shows erythrocyte-containing lumina lined by a single layer of bland, flattened endothelial cells, without cytologic atypia or mitotic activity. Scale bar = 0.05 mm. (C) D2-40 highlights background lymphatic vessels but is negative in the lesional vascular channels.

Imaging and pathology were considered concordant. Given the absence of symptoms, small lesion size, and benign pathology without atypia, no surgical excision was performed. The patient was returned to routine annual screening mammography, with the next examination scheduled at 12 months.

## Discussion

Breast hemangiomas have been reported across a broad age range, from 1.5 to 82 years [3]. Capillary hemangiomas have also been described in older women. Lee et al. reported 2 subcutaneous capillary hemangiomas in a 72-year-old woman, including an incidentally detected, partly obscured oval mammographic mass [4]. The present case is therefore not unique solely because of the patient's age. Rather, its educational value lies in the detection of a newly developed, subcentimeter mass on screening mammography in an asymptomatic 74-year-old woman and in the imaging-pathology concordance that supported conservative management following benign core needle biopsy.

Mammographically, hemangiomas may appear as oval or lobulated masses and may be partially obscured by adjacent tissue. Calcifications are not required. On ultrasound, they may be hypoechoic or heterogeneous and frequently demonstrate posterior acoustic enhancement [[1], [2], [3]]. Color Doppler appearances are variable, ranging from absent or minimal internal flow to more conspicuous vascularity [2,3,5]. In the present case, Doppler demonstrated limited internal flow associated with a feeding vessel. When magnetic resonance imaging is performed, reported findings include high T2 signal and persistent or plateau enhancement, although MRI was not required in this case [2,6,7].

The main diagnostic concern is low-grade angiosarcoma, which may overlap with benign vascular lesions on imaging and may occasionally be under-sampled on core biopsy. Pathology should therefore be interpreted together with imaging morphology, lesion size, interval change, and clinical context [8]. In this case, the biopsy showed well-formed, erythrocyte-containing capillary spaces lined by bland endothelial cells without atypia or mitotic activity. D2-40 negativity in the lesional channels argued against lymphatic differentiation, while the blood-vascular nature of the lesion was evident morphologically. No histologic features of angiosarcoma were identified in the sampled tissue. Because core biopsy samples only part of a lesion, this finding should not be interpreted as definitive exclusion of angiosarcoma when imaging or clinical findings are discordant.

Management of breast capillary hemangioma in older patients should be individualized and guided primarily by imaging-pathology concordance rather than age alone. When core biopsy demonstrates a benign vascular lesion without atypia and adequately explains the imaging finding, surgical excision is not routinely indicated [3]. In the present case, because the patient was asymptomatic and the small lesion had benign concordant pathology, no excision was performed; she was returned to routine annual mammographic screening, with the next examination scheduled at 12 months rather than short-interval follow-up. Repeat sampling or surgical excision should be considered if imaging and pathology are discordant, the lesion enlarges, symptoms develop, or the specimen is limited or shows atypical vascular proliferation. Comorbidities, procedural risk, and patient preference should also be incorporated into decision-making.

In conclusion, capillary hemangioma may present as a new mass on screening mammography even when its imaging morphology is relatively benign. Interval development may appropriately prompt biopsy. In an asymptomatic older patient, benign concordant core-biopsy findings can support conservative management without surgical excision, whereas discordance, growth, symptoms, or atypical or limited sampling should prompt further evaluation.

## Patient consent

Written informed consent was obtained from the patient for publication of this case report and any accompanying images. A copy of the written consent is available for review by the Editor-in-Chief of this journal upon request.
